# Yanghe Huayan decoction inhibits the capability of trans-endothelium and angiogenesis of HER2+ breast cancer via pAkt signaling

**DOI:** 10.1042/BSR20181260

**Published:** 2019-02-15

**Authors:** Xiao-Fei Liu, Jing-Wei Li, Hong-Zhi Chen, Zi-Yuan Sun, Guang-Xi Shi, Jian-Min Zhu, Ai-Li Song, Ying Wang, Xiang-Qi Li

**Affiliations:** 1 First Clinical College, Shandong University of Traditional Chinese Medicine, Jinan, Shandong, China; 2 Department of Breast Surgery, Affiliated Hospital of Shandong University of Traditional Chinese Medicine, Jinan, Shandong, China; 3 College of Traditional Chinese Medicine, Shandong University of Traditional Chinese Medecine, Jinan, Shandong, China; 4 Department of Breast Surgery, Affiliated Hospital of Taishan Medical University, Tai’an, Shandong, China; 5 Department of Cardiovascular Surgery, The Affiliated Hospital of Soochow University, Suzhou, China

**Keywords:** angiogenesis, breast cancer, pAkt signaling, trans-endothelium, Yanghe Huayan Decoction

## Abstract

**Background**: Yanghe Huayan Decoction (YHD), a traditional Chinese medicine, is one of the most common complementary medicine currently used in the treatment of breast cancer (BC). It has been recently linked to suppress precancerous lesion and tumor development. The current study sought to explore the role of YHD on trans-endothelium and angiogenesis of BC. **Methods:** HER2+ BC cells were treated with YHD, Trastuzumab, or the combination *in vitro* and *in vivo* to compare the effects of them on trans-endothelium and angiogenesis features. The present study also investigated the potential molecular mechanism of YHD in inhibiting angiogenesis of BC. **Results**: YHD significantly suppressed the invasion and angiogenesis of BC cells via elevated pAkt signaling. Administration of YHD *in vivo* also strikingly repressed angiogenesis in tumor grafts. **Conclusion:** YHD could partially inhibit and reverse tumorigenesis of BC. It also could inhibit Akt activation and angiogenesis *in vitro* and *in vivo*. Its effect was superior to trastuzumab. Thus it was suitable for prevention and treatment of BC.

## Introduction

Breast cancer (BC) is one of the leading malignancies in women, accounting for ∼25% cases worldwide [1]. It results in ∼1.68 million cases and 522000 deaths per year [1]. The prognosis of BC is really poor and tumor metastasis and recurrence are considered to be the most important determinants for treatment failure. Although current treatment options are available for early BC, standard therapeutic strategies for advanced BC cannot circumvent exacerbation of patients [2]. Genetic rearragement during tumor metastasis enables cancer cells to acquire resistance to different chemo- and radiotherapy, accounting for the high mortality of advanced BC [3]. Collectively, it is urgent to develop more effective therapeutic strategies for the affected patients.

Traditional Chinese medicines in complementary therapies have been considered as a more ‘natural’ approach to improve hypoimmunity and acquire high quality of life, particularly for patients with advanced cancer [4]; 2500 years ago, a canonical medical manual in China— Yellow Emperor’s Classic of Internal Medicine described the first phytotherapeutic treatment of BC [4]. Generous studies confirmed the notable effects of traditional Chinese medicine in BC management. For instance, Huaier (*Trametes robiniophila Murr*) sensitized BC cells to radiotherapy via cell cycle arrest [5]. A population-based study revealed that adjunctive Traditional Chinese medicine treatment might lower the mortality of patients with advanced BC [6].

Yanghe Huayan Decoction (YHD), is composed of eight herbs, including Lu jiao shuang (*Cornu cervi degelatinatum*), Shu di (*Rehmannia glutinosa*), Rou gui (*Cinnamomum cassia presl*), Bai jie zi (*Semen brassicae*), E zhu (*Rhizoma curcumae phaeocaulis*), Shan ci gu (*Pseudobulbus Cremastrae Seu Pleiones*), Zhe bei mu (*Bulbus Fritillariae Thunbergii*), and Gan cao (licorice) [7]. Although the active ingredients and pharmacological mechanism of YHD in BC treatment remain unclear, extract from certain ingredients of YHD present antitumor activity. For example, Lu jiao extract significantly suppressed hyperplasia of mammary glands in rats, the pathological change related to BC [8]. Extract of Bai jie zi—sinapine exhibited growth-suppressive activity and reversed multidrug resistance in MCF/dox cancer cells [9]. Whereas E zhu extracts presented cytotoxic effect on BC cells and induced ROS formation and cell apoptosis [10,11]. The current study demonstrated that YHD exhibited antitumor activity against BC xenografts in nude mice. YHD showed potent cytotoxicity against BC cells *in vitro* via activation of pAkt signaling. The present findings provided a clear rationale to explore the treatment protocols of using YHD alone or in combination with chemotherapeutic agents for BC patients and the potential functional mechanism of its active principles.

## Materials and methods

### Herbs and preparation of aqueous extracts

All herbs in the YHD formula were obtained from the Affiliated Hospital of Shandong University of Traditional Chinese Medicine. The dry weights of the eight herbs were mixed at fixed ratios (Lu jiao shuang:Shu di:Rou gui:Bai jie zi:E zhu:Shan ci gu:Zhe bei mu:Gan cao = 4:3:2:1:4:5:3:2). All herbs (72 g) were ground to a powder and extracted twice by 1 l ddH_2_O, 100°C. Then the solution was concentrated to 200 ml. The concentration of YHD was 270 mg/ml. The supernatant was filtered through 0.45-μm filters, concentrated and spray-dried to generate a brown fine powder. This powder was dissolved in PBS at the desired concentration in following experiments.

### Dose justification with cell counting kit-8 assay

Dose justification of YHD on MM-453 cells was performed using a Cell Counting Kit-8 (CCK-8) (Dojindo, Tokyo, Japan). Cells were seeded in a 96-well plate with 5 × 10^3^ cells/well in 100 μl medium. Then they were treated with YHD at different doses. Each group had six repeated wells. Cell viability was tested 24 h later with 2 h incubation with 10 μl CCK-8 reagent/well (inhibition ratio = 1 − cell viability%). Absorbance was examinated at 450 nm on a microplate reader. Finally, YHD IC_25_ dose was 200 μg/ml, and IC_50_ concentration was 367 μg/ml (Supplementary Figure S1).

**Figure 1:**
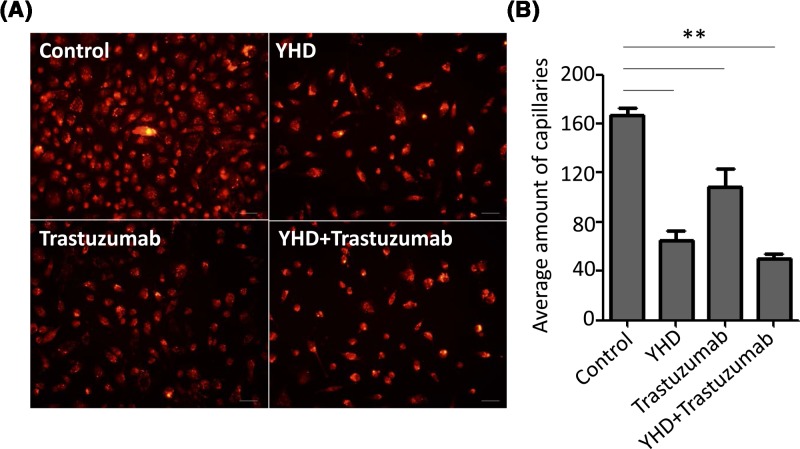
The effect of YHD, Trastuzumab, or their combination on trans-endothelium capability of MM-453 cells (**A**) Fluorescence images of MM-453 cells trans-endothelium treated by YHD, Trastuzumab, or their combination. (**B**) Quantitation analysis of (A). All experiments were replicated for three times. Data were presented as mean ± S.D. Scale bar = 100 μm. **P<0.01.

### Cell line, mice, and BC xenograft models

The HER2+ BC MDA-MB-453 (MM-453) cell line and human umbilical vein endothelial cells (HUVECs) were obtained from the American Type Culture Collection (Manassas, VA, U.S.A.). The cells were cultured in DMEM supplemented with 10% FBS and EGM-2 BulletKit medium (Clonetics, MD, U.S.A.), respectively. Trastuzumab was purchased from Roche Pharma AG (Grenzach-Wyhlen, Germany). The animal experimental protocols was approved by the ethics committee of Shandong University of Traditional Chinese Medicine. Female pathogen-free nude mice (nu/nu, age: 5–6 weeks; weight: 20–25 g) were obtained from Laboratory Animal Center of Shandong University of Traditional Chinese Medicine (Jinan, Shandong, China). To establish HER2+ BC xenograft models, 5 × 10^6^ MM-453 cells in 100 μl medium were subcutaneously injected to the right flank of each mouse. All mice were monitored for body weight, activity, and tumor volume. While the tumor volume reached ∼50 mm^3^ (1/2L*W^2^), the xenograft models were considered successfully constructed. Xenograft models were randomly classified into four groups: Control, YHD, trastuzumab, YHD+trastuzumab (*n*=10/group). Trastuzumab was intraperitoneally injected at 2 mg/kg every 3 days; YHD was intragastrically administrated at 50 mg/kg everyday. Control group only received the vehicle. Experiments were performed for 4 weeks.

### Trans-endothelium and tube formation assay

Cell trans-endothelium measurements were performed using 24-well Transwell® units (8.0 μm pore, Costar Corning, NY, U.S.A.). In brief, HUVECs (5 × 10^3^) were seeded on upper chamber and confluency was excellent 1 day before experiment. Then transwell assay was performed in different groups. Dil (Beyotime, Shanghai, China) prestained MM-453 cells (1 × 10^3^) in 100 μl DMEM with 5% FBS was added to upper chambers, and 500 μl of DMEM with 10% FBS was added to the lower chambers, with treatment of YHD (200 μg/ml, at IC_25_ dose, Supplementary Figure S1), trastuzumab (50 μg/ml, at IC_25_ dose) or combination in upper chamber. The plate was incubated at 37°C, 5% CO_2_ for 24 h. The non-migrated MM-453 cells in the insert were wiped away and the trans-endothelium cells were counted under an inverse fluorescence microscope (Olympus IX51, Tokyo, Japan). Cells cross through the HUVECs chamber were quantitated by counting ten independent visual fields to calculate the average trans-endothelium ratio. Each assay were performed in triplicate. In tube formation assay, HUVECs were co-cultured with MM-453 cells treated with YHD (200 μg/ml), trastuzumab (50 μg/ml), or combination for 3 days. Basement Membrane Matrigel (BD Biosciences, San Jose, CA, U.S.A.) (150 μl) was added to each well of a 96-well plate and coagulated for 30 min at 37°C. Co-cultured HUVECs (2 × 10^3^ cells) were seeded on the surface of Matrigel. Cells were incubated for 12 h in DMEM containing 5% FBS. Morphological changes were observed and photographed by an inverse fluorescence microscope (Olympus IX51, Tokyo, Japan). Quantitative analysis of tube length was conducted using Image-Pro Plus 6.0 (Media Cybernetics, Silver Spring, MD, U.S.A.).

### Immunofluorescence assay for angiogenesis *in vivo*

Thirty minutes before killing, the xenograft models (*n*=10/group) were intratumorally injected with unconjugated Griffonia (Bandeiraea) simplicifolia lectin I (Vector lab, U.S.A.) to mark the newborn capillaries. Xenograft tumors were fixed in 4% paraformaldehyde and embedded in paraffin. Serial cross-sections (5 μm) were prepared, dewaxed, and hydrated. Then they were blocked and incubated with Anti-Griffonia (Bandeiraea) Simplicifolia Lectin I (1:100, Vector lab, U.S.A.), Alexa Fluor® 594 Donkey anti-Goat IgG antibody (1:1000, Abcam, U.S.A.) and DAPI. The immunofluorescence images for lectin markered capillaries in tumors were digitally captured on a Carl Zeiss LSM880 laser scanning confocal microscope (Carl Zeiss, Jena, Germany). Capillary numbers in xenograft tumors with different treatment were quantitatively analyzed from ten samples per group using Image ProPlus 6.0 software (Media Cybernetics, Silver Spring, MD, U.S.A.).

### Western blot for mitogen-activated protein kinase and Akt detection

Cells (1 × 10^7^) in each group were broken using RIPA buffer. Protein was extracted, and separated on 12% SDS/PAGE. Then it was transferred to PVDF membranes (Millipore, Bedford, U.S.A.), incubated with antibodies of mitogen-activated protein kinase (MAPK), Akt, pAkt (Ser^473^), GAPDH (Santa Cruz Biotechnology, 1:1000). Finally, the membranes were visualized using ECL substrate solution (Bio-Rad, U.S.A.) and examined by Bio-Rad™ gel imaging analysis system (Bio-Rad, U.S.A.). GAPDH was an internal control.

### Immunohistochemistry for pAkt level evaluation *in vivo*

The Akt phosphorylation in xenograft tumors (*n*=10/group) with different treatments was detected by immunohistochemistry. First, the tumors were dissected from anesthetic animals and fixed in 4% paraformaldehyde for 4 h. Second, they were paraffin-embedded and cut into 5-μm sections. Rabbit monoclonal antibody against pAkt (1:100, Santa Cruz Biotechnology) was applied for immunostaining according to the manufacturer’s instructions (Servicebio, Wuhan, China). The images was independently evaluated by two researchers (X.L. and J.L.) under a phase-contrast microscope (Olympus BX51, Tokyo, Japan). They were blinded to the groups of nude mice. Quantitative analysis was performed with the Image-Pro Plus 6.0 software (Media Cybernetics, Silver Spring, MD, U.S.A.). The stained sections were examined at 200× magnification. Ten representative staining fields/each section were evaluated to generate the Mean Integral Optical Density (MIOD). MIOD represents the intensity of staining signals as measured positive pixels. The negative control was used for background subtraction.

### Statistical analysis

One-way ANOVA was used to detect the differences of continual variables amongst four groups. All statistical tests were two-sided and performed using SPSS 18.0 for Windows (SPSS, Chicago, III). *P*<0.05 was considered significant.

## Results

### YHD inhibited trans-endothelium and angiogenesis of BC cells

**Figure 2:**
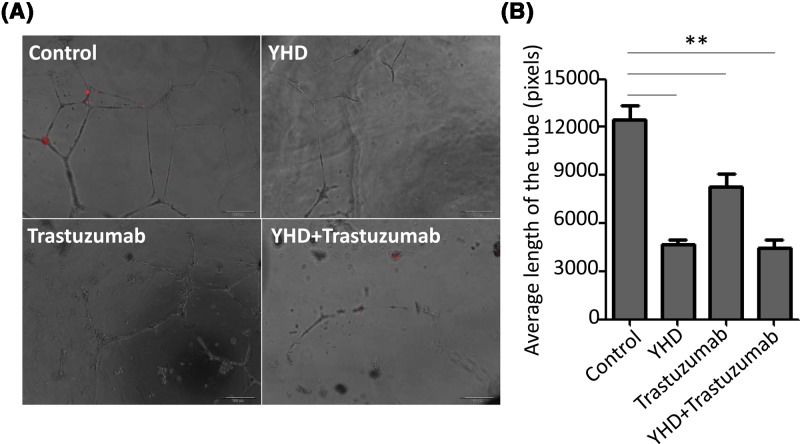
The impact of YHD, Trastuzumab, or their combination on angiogenesis (**A**) Bright-field images of HUVEC cells co-cultured with MDA-MB-453 cells treated by YHD, Trastuzumab, or their combination. (**B**) Quantitation analysis of (A). All experiments were replicated for three times. Data were presented as mean ± S.D. Scale bar = 200 μm. **P<0.01.

For BC, the pathological progression including atypical hyperplasia of mammary gland, tumorigenesis, trans-endothelium, infiltration, and angiogenesis. Amongst them, trans-endothelium and angiogenesis are most essential processes of BC because they facilitate the aggressiveness of BC [12,13]. To evaluate the impact of YHD, trastuzumab, or the combination on trans-endothelium and angiogenesis of BC cells, we plated HUVECs on upper chamber and performed transwell assay in different groups. The data indicated that YHD significantly inhibited cells crossing through HUVEC-plated upper chamber by 61.15% (Control: 167 ± 10 cells, YHD: 65 ± 14 cells, *P*=0.000), and the inhibition of combination was the most effective (inhibited by 70.06%, Control: 167 ± 10 cells, YHD + trastuzumab: 50 ± 6 cells, *P*=0.000) (Figure 1). Tube formation assay further proved that YHD significantly suppressed the pro-angiogenic effect of HER2+ BC cells by 62.60% (Control: 12488 ± 1458 pixels, YHD: 4671 ± 502 pixels, *P*=0.000). Trastuzumab treatment notably decreased the tube length by 33.46% (Control: 12488 ± 1458 pixels, trastuzumab: 8318 ± 1310 pixels, *P*=0.021). The effect of YHD was significantly superior to that of trastuzumab in angiogenesis (YHD: 4671 ± 502 pixels, trastuzumab: 8318 ± 1310 pixels, *P*=0.011). However, the combination reduced the pro-angiogenic effect of BC cells by 64.53% (Control: 12488 ± 1458 pixels, YHD+trastuzumab: 4429 ± 966 pixels, *P*=0.001) (Figure 2). Of note, there was no obvious change in the tube length between YHD and combination groups.

### YHD inhibited angiogenesis of BC *in vivo*

**Figure 3:**
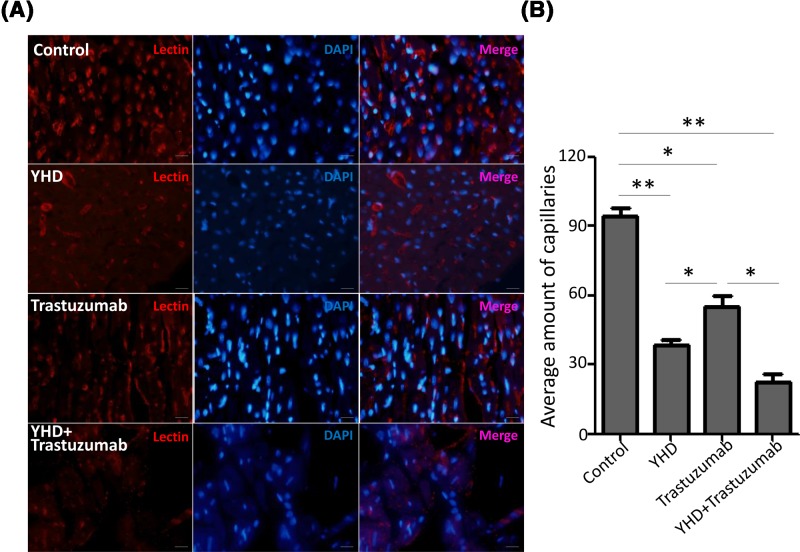
The impact of YHD, Trastuzumab or their combination on angiogenesis *in vivo* (**A**) The representative immunofluorescence graphs for lectin expression in xenograft tumors of nude mice treated with YHD, Trastuzumab, or their combination. (**B**) Quantitation analysis of (A). Data were presented as mean ± S.D. Scale bar = 100 μm. *P<0.05, **P<0.01.

We further constructed xenograft models using nude mice bearing BC tumors to evaluate the effect of YHD on tumor angiogenesis *in vivo*. The incidence of tumor in animal models was ∼80%. Successful xenograft models were intragastrically administrated with YHD, intravenously injected with trastuzumab, or treated with combination. Four weeks after treatment, mice were intratumorally injected with lectin directly labeling antibody and killed. Frozen sections of xenograft tumors were stained with DAPI before immunofluorescence analysis. The result revealed that YHD or trastuzumab alone strikingly inhibited angiogenesis of xenograft tumors (Control: 94 ± 7 capillaries, YHD: 39 ± 4 capillaries, *P*=0.000) (Control: 94 ± 7 capillaries, trastuzumab: 68 ± 12 capillaries, *P*=0.034). The combination also notably reduced the number of capillaries in xenograft models (Control: 94 ± 7 capillaries, YHD+trastuzumab: 22 ± 6 capillaries, *P*=0.000), while the effect of YHD was better than that of trastuzumab (YHD: 39 ± 4 capillaries, trastuzumab: 68 ± 12 capillaries, *P*=0.016) (Figure 3). These findings were consistent with data of angiogenesis *in vitro*.

### YHD suppressed the activation of Akt signaling of BC cells *in vitro* and *in vivo*

**Figure 4:**
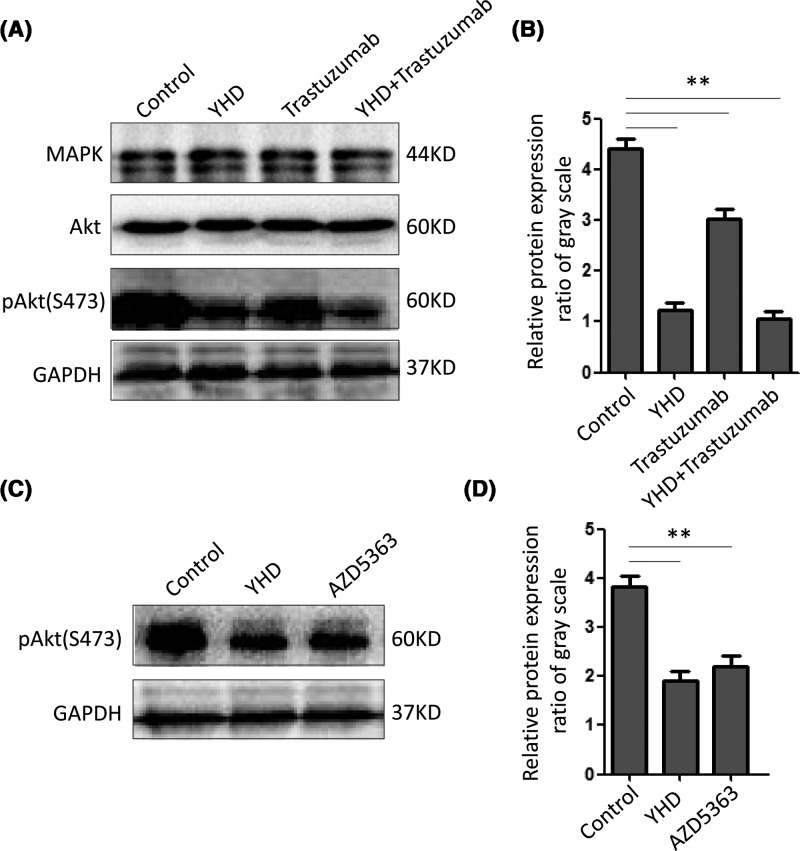
The expression of MAPK or Akt signaling molecules in BC cells with treatment of YHD, Trastuzumab, or their combination (**A**) Western blot assay of MAPK, Akt, pAkt (S473) in BC cells of different groups and relative quantitation analysis (**B**). (**C**) The expression of pAkt (S473) in BC cells of YHD or AZD5363 treatment and quantitation analysis (**D**). All experiments were performed in triplicate. Data were presented as mean ± S.D. **P<0.01.

**Figure 5:**
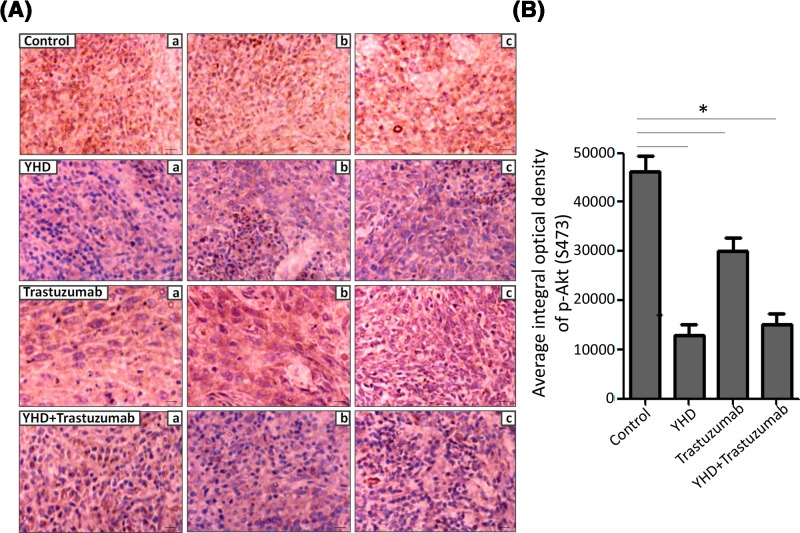
The pAkt (S473) level in tumors with treatment of YHD, Trastuzumab, or their combination (**A**) The representative images of immunohistochemistry assay for pAkt level in tumor of mice treated with YHD, Trastuzumab, or their combination. (**B**) Quantitation analysis of (A). Data were presented as mean ± S.D. Scale bar = 100 μm. *P<0.05.

To investigate the potential mechanism by which YHD suppressed tumor trans-endothelium and angiogenesis, we examined the expression of relevant molecules in tumor metastasis and angiogenesis. As shown in Figure 4, Western blot assay suggested that the level of pAkt (Ser^473^) was significantly lower in YHD group than that in trastuzumab group (YHD: 1.23 ± 0.25 folds, trastuzumab: 3.03 ± 0.32 folds, *P*=0.002). But there was no striking discrimination in pAkt level between YHD and combination groups (YHD: 1.23 ± 0.25 folds, YHD+trastuzumab: 1.07 ± 0.25 folds, *P*=0.463). The data suggested that using trastuzumab did not affect the function of YHD on phosphorylation of Akt in BC cells. The immunohistochemistry assay *in vivo* also confirmed this phenomenon (YHD: 12848 ± 3803 pixels, trastuzumab: 30113 ± 4612 pixels, *P*=0.007) (Figure 5). In addition to inhibitor of Akt phosphorylation-AZD5363, the impact was similar to that of YHD (YHD: 1.90 ± 0.36 folds, AZD5363: 2.20 ± 0.36 folds, *P*=0.366) (Figure 4). Besides, there was no obvious change in MAPK and Akt expression amongst YHD, trastuzumab, YHD+trastuzumab groups (Figure 4). These results supported the hypothesis that pAkt signaling was involved in YHD effect on malignant transformation of BC.

### Administration of YHD in combination with Trastuzumab was more effective than the gene therapy alone

To investigate the protocol of using YHD alone or in combination with genetherapeutic agents for patients, we compared the influence of YHD, or combination with trastuzumab on trans-endothelium and angiogenesis of BC. The observations revealed that the combination was more effective in inhibiting the capability of trans-endothelium than that of trastuzumab alone for HER2+ BC cells (Control: 167 ± 10 cells, trastuzumab: 109 ± 25 cells, YHD + trastuzumab: 50 ± 6 cells, *P*=0.016). Similarly, tube formation assay further proved that the combination was more significant in suppressing the pro-angiogenic effect of HER2+ BC cells (Control: 12488 ± 1458 pixels, trastuzumab: 8318 ± 1310 pixels, YHD+trastuzumab 4429 ± 966 pixels, *P*=0.000) (Figure 2). For *in vivo* assay, the effect of combination was strikingly superior to that of trastuzumab in inhibiting angiogenesis of xenograft tumors (Control: 94 ± 7 capillaries, trastuzumab: 68 ± 12 capillaries, YHD+trastuzumab: 22 ± 6 capillaries, *P*=0.004) (Figure 3). In Western blot and immunohistochemistry detection for Akt phosphorylation, the combination treatment had the most potent effect in down-regulating pAkt level (Western blot for *in vitro* assay: Control: 4.40 ± 0.36 folds, trastuzumab: 3.03 ± 0.32 folds, YHD+trastuzumab: 1.07 ± 0.25 folds, *P*=0.001) (Figure 4) (immunohistochemistry for *in vivo* assay: Control: 46163 ± 5577 pixels, trastuzumab: 30113 ± 4612 pixels, YHD+trastuzumab: 15107 ± 3722 pixels, *P*=0.012) (Figure 5). Taken together, the effect of YHD and trastuzumab combination was significantly superior to that of the genetherapeutic agent.

## Discussion

According to the mindset of traditional Chinese medicine, cancer is a complex disease formed because of the imbalance of body homeostasis [14]. Folk Chinese traditional medicine attributes BC to the accumulation of heat, swelling, and blood stasis. This pathological process is considered as ‘breast rock’ in ancient Chinese medical literature [4]. Recently, pre-clinical and clinical studies have demonstrated that traditional Chinese medicine combined with routine Western medicine therapy, chemotherapy, and radiotherapy, can provide more benefits to tumor patients. Traditional Chinese medicine increases the sensitivity of anticancer drugs, reduces the side effects, and improves the quality of life of patients [15,16]. Ruanjian Sanjie decoction, another traditional Chinese medicine used in BC treatment, was found inducing BC cells apoptosis via Bcl-2/survivin signaling [17]. In our study, we explored the potential mechanism of a newly reported traditional Chinese medicine—YHD inhibiting the development of HER2+ BC, and verified the efficiency of YHD combined with trastuzumab on inhibiting angiogenesis and progression of advanced BC. To the best of our knowledge, this is the first study declaring the effect of YHD and trastuzumab combination in BC.

YHD, a traditional Chinese medicine used in treatment for advanced BC, was first reported in our previous study [7]. Although the active ingredients and pharmacological mechanism of YHD in BC treatment remained unclear, extract from certain ingredients of YHD presented antitumor activity [8,9]. For BC, trans-endothelium and angiogenesis are most essential proceses of BC because they facilitate the aggressiveness of BC [12,13,18]. To evaluate the impact of YHD, trastuzumab, or the combination on trans-endothelium and angiogenesis of BC cells, we performed transwell and tube formation assays in different groups. We found that YHD or trastuzumab significantly inhibited cells crossing through HUVEC-plated upper chamber by 61.15 or 34.85%, respectively. The inhibition of combination was most effective (inhibited by 70.06%) (Figure 1). Tube formation assay further proved that YHD or trastuzumab significantly suppressed the pro-angiogenic effect of HER2+ BC cells by 62.60 or 33.40%, respectively. The combination reduced the pro-angiogenic activity of BC cells by 64.53% (Figure 2). The *in vivo* angiogenesis experiments was in concordance with the data *in vitro*. These findings suggested that YHD significantly suppressed the malignant formation of HER2+ BC both *in vitro* and in xenograft tumors. The combination exhibited better effects than YHD or trastuzumab alone. Patients with HER2+ BC might benefit more from the combined Chinese and western medicine strategy—YHD and routine genetherapy agent, trastuzumab.

PI3K/Akt signaling plays a pivotal role in metastasis and angiogenesis of tumor development [19–21]. Cancer development depends on vigorous glycolysis and glucose utilization, which resulted from activation of Akt and up-regulation of pyruvate kinase M2 (M2-PK). As a valuable prognostic marker in BC, the expression of pAkt was always analyzed in BC samples [22]. In order to evaluate the potential mechanism by which YHD suppressing tumor trans-endothelium and angiogenesis, we examined the expression of relevant molecules in tumor metastasis and angiogenesis. The observations confirmed that there was no obvious change in MAPK and Akt expression amongst YHD, trastuzumab, YHD+trastuzumab groups (Figure 4). However, phosphorylation level of Akt (Ser^473^) in BC cells was down-regulated most intensely with combination treatment, followed by YHD, trastuzumab. In addition to inhibitor of Akt phosphorylation- AZD5363, the impact was similar to that of YHD. The data suggested that YHD and the combination might suppress tumor aggressiveness via interfering with the pAkt signaling in HER2+ BC cells. The *in vivo* immunohistochemistry assay of xenograft tumors also confirmed this phenomenon (Figure 5). These results supported the hypothesis that pAkt signaling was substantially involved in potential mechanism of YHD and trastuzumab combination inhibiting malignant transformation such as trans-endothelium and angiogenesis of BC.

**Figure 6:**
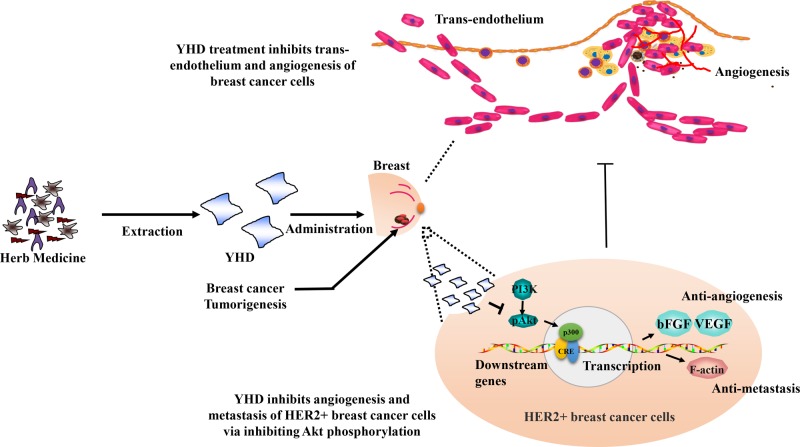
Schematic diagram for the potential functional mechanism of YHD in angiogenesis and metastasis of HER2+ BC cell YHD might inhibit cancer cell capability of trans-endothelium and angiogenesis via suppressing Akt signal activation, and further inhibit the development of HER2+ BC.

In conclusion, the present study confirmed that the traditional Chinese medicine—YHD shows potent anti-HER2+ BC activity *in vitro* and *in vivo*. And the effect of combination was superior to that of the genetherapeutic agent—trastuzumab alone. YHD significantly inhibited trans-endothelium properties of HER2+ BC cells, further suppressing the pro-angiogenic activity both *in vitro* and *in vivo* via activating Akt signaling (Figure 6). Additionally, considering that YHD administration was well tolerated, the combination of YHD and genetherapeutic drugs exhibited a synergistic effect *in vivo*, particularly in advanced BC xenograft models. More investigations into the clinical implication of YHD in advanced BC treatment and the characterization of its active principles were required.

## Availability of data and material

Please contact authors for data requests.

## Supporting information

**Supplementary Figure 1:** 

## Abbreviations

BC: breast cancer
CCK-8: Cell Counting Kit-8
HUVEC: human umbilical vein endothelial cell
MAPK: mitogen-activated protein kinase
MIOD: mean integral optical density
YHD: Yanghe Huayan decoction
