# Correction: Yanghe Huayan Decoction inhibit the capability of trans-endothelium and angiogenesis of HER2+ breast cancer via pAkt signaling

**DOI:** 10.1042/BSR-2018-1260_COR

**Published:** 2023-09-06

**Keywords:** angiogenesis, breast cancer, pAkt signaling, trans-endothelium, Yanghe Huayan Decoction

The authors of the original article “Yanghe Huayan Decoction inhibit the capability of trans-endothelium and angiogenesis of HER2+ breast cancer via pAkt signaling” (DOI: 10.1042/BSR20181260) would like to correct Figure 1.

**Figure 1:**
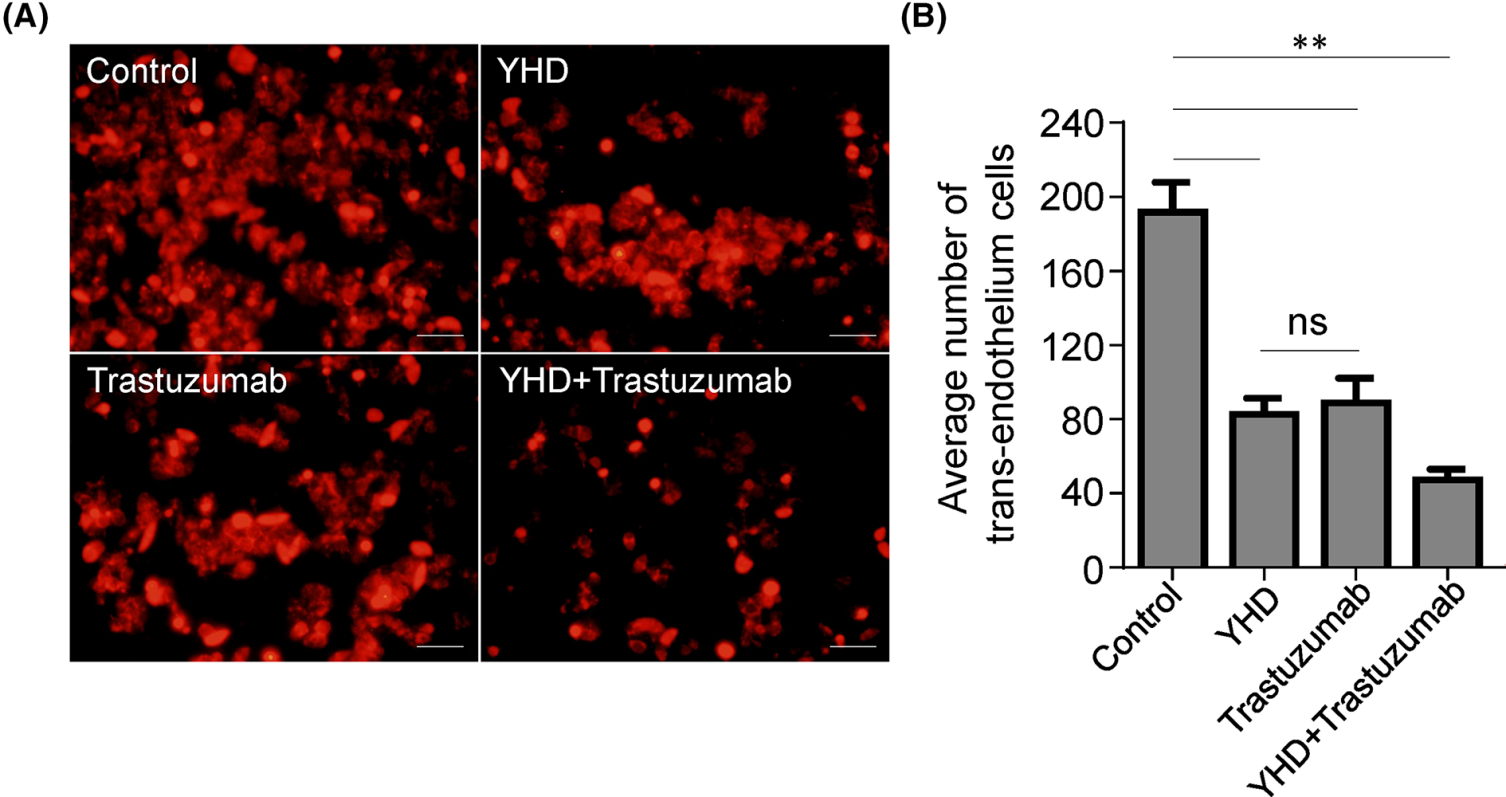
The effect of YHD, Trastuzumab, or their combination on trans-endothelium capability of MM-453 cells (**A**) Fluorescence images of MM-453 cells trans-endothelium treated by YHD, Trastuzumab, or their combination. (**B**) Quantitation analysis of (**A**). All experiments were replicated for three times. Data were presented as mean ± S.D. Scale bar = 100 μm. **P<0.01.

After publication the authors identified that the data had been incorrectly calculated, the current result could not be generated from the raw data and that the title for the Histogram was incorrect.

In addition, the following text from the first paragraph of the Results section: The data indicated that YHD significantly inhibited cells crossing through HUVEC-plated upper chamber by 61.15% (Control: 167 ± 10 cells, YHD: 65 ± 14 cells, P = 0.000), and the inhibition of combination was the most effective (inhibited by 70.06%, Control: 167 ± 10 cells, YHD + trastuzumab: 50 ± 6 cells, P = 0.000) (Figure 1).

Should instead read: The data indicated that YHD significantly inhibited cells crossing through HUVEC-plated upper chamber by 56.70% (Control: 194±25 cells, YHD: 84±12 cells, P = 0.000), and the inhibition of combination was the most effective (inhibited by 74.23%, Control: 194±25 cells, YHD+ trastuzumab: 50±8 cells, P = 0.000). However, there was no obvious difference of tans-endothelium capability between YHD and trastuzumab groups (YHD: 84±12 cells, trastuzumab: 91±20 cells, P = 0.660) (Figure 1).

The requested correction has been assessed and agreed by the Editorial Board. The authors declare that these corrections do not change the results or conclusions of their paper.

