# Supplementary figures and images for: Yanghe Huayan decoction inhibits the capability of trans-endothelium and angiogenesis of HER2+ breast cancer via pAkt signaling
Source: Biosci Rep. 2019 Feb 15;39(2):BSR20181260. doi: 10.1042/BSR20181260 (PMC6379224; doi:10.1042/BSR20181260)

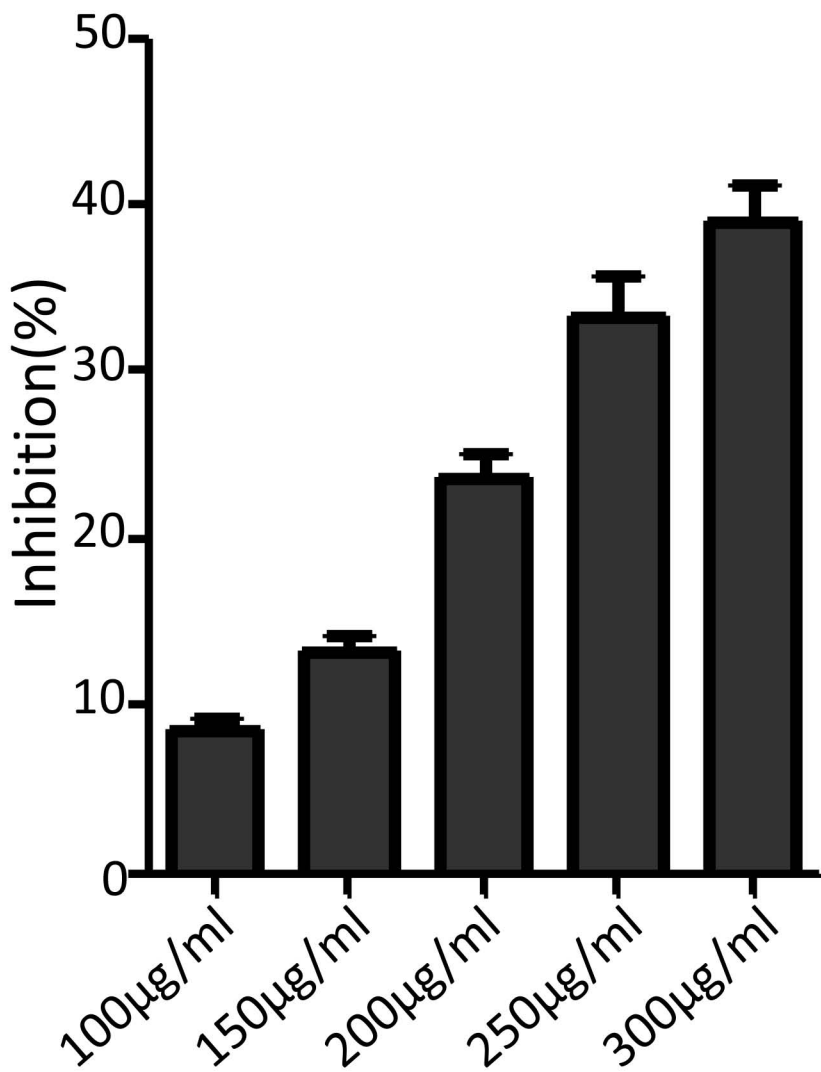

Supplement: Supplementary file 1 [file bsr-39-bsr20181260_Supp1.pdf]
